# Redox-dependent thiol modifications: implications for the release of extracellular vesicles

**DOI:** 10.1007/s00018-018-2806-z

**Published:** 2018-03-28

**Authors:** Birke J. Benedikter, Antje R. Weseler, Emiel F. M. Wouters, Paul H. M. Savelkoul, Gernot G. U. Rohde, Frank R. M. Stassen

**Affiliations:** 1 Department of Medical Microbiology, NUTRIM School of Nutrition and Translational Research in Metabolism, Maastricht University Medical Center, PO Box 5800, 6202 AZ Maastricht, The Netherlands; 2 Department of Respiratory Medicine, NUTRIM School of Nutrition and Translational Research in Metabolism, Maastricht University Medical Center, PO Box 5800, 6202 AZ Maastricht, The Netherlands; 3 Department of Pharmacology and Toxicology, NUTRIM School of Nutrition and Translational Research in Metabolism, Maastricht University, PO Box 616, 6200 MD Maastricht, The Netherlands; 4 Department of Medical Microbiology and Infection Control, VU University Medical Center, P.O. Box 7057, 1007 MB Amsterdam, The Netherlands; 5 Medical Clinic I, Department of Respiratory Medicine, Goethe University Hospital, Frankfurt/Main, Germany

**Keywords:** Exosomes, Microvesicles, Sulfhydryl groups, Redox environment, Chronic inflammation, *N*-acetyl-l-cysteine

## Abstract

Extracellular vesicles (EVs), including microvesicles and exosomes, are emerging as important regulators of homeostasis and pathophysiology. During pro-inflammatory and pro-oxidant conditions, EV release is induced. As EVs released under such conditions often exert pro-inflammatory and procoagulant effects, they may actively promote the pathogenesis of chronic diseases. There is evidence that thiol group-containing antioxidants can prevent EV induction by pro-inflammatory and oxidative stimuli, likely by protecting protein thiols of the EV-secreting cells from oxidation. As the redox state of protein thiols greatly impacts three-dimensional protein structure and, consequently, function, redox modifications of protein thiols may directly modulate EV release in response to changes in the cell’s redox environment. In this review article, we discuss targets of redox-dependent thiol modifications that are known or expected to be involved in the regulation of EV release, namely redox-sensitive calcium channels, *N*-ethylmaleimide sensitive factor, protein disulfide isomerase, phospholipid flippases, actin filaments, calpains and cell surface-exposed thiols. Thiol protection is proposed as a strategy for preventing detrimental changes in EV signaling in response to inflammation and oxidative stress. Identification of the thiol-containing proteins that modulate EV release in pro-oxidant environments could provide a rationale for broad application of thiol group-containing antioxidants in chronic inflammatory diseases.

## Extracellular vesicles

Extracellular vesicles (EVs) are small membrane-surrounded vesicles that are secreted by virtually all cell types and that have been detected in various body fluids including plasma [1], urine [2] and bronchoalveolar lavage fluid [3]. These EVs carry a complex cargo composed of proteins, nucleotides and lipids, among others [4]. They can exert multiple biological effects, either by interacting with or being taken up by target cells, or in the extracellular space [5]. As EVs are released under physiological and stress conditions, they have been attributed various functions in homeostasis as well as pathology [5].

### EV biogenesis and uptake

In recent years, it has become clear that EVs are a heterogeneous population of membrane-delimited structures that emerge from distinct biogenetic pathways, and that have diverse biophysical and biochemical properties [6, 7]. This review focuses on two major EV types, exosomes and microvesicles, whose properties are summarized in Table 1. Several recent review articles provide a detailed overview of the current knowledge on EV biogenesis [8–10] and uptake [11, 12]. Therefore, these processes will be described only briefly and non-comprehensively, to provide the necessary context for later sections of this article. Exosome biogenesis takes place in late endosomes, which transform into multivesicular bodies (MVBs) by inward budding of their membrane to form small intraluminal vesicles (ILVs). These MVBs can then fuse with the plasma membrane and release their ILVs to the extracellular space as exosomes. In contrast to exosomes, microvesicles are released from the cell by outward budding of the plasma membrane followed by membrane fission. The membrane rearrangements that occur during exosome and microvesicle biogenesis are regulated by energy-dependent enzymes, including Rab GTPases and the endosomal sorting complex required for transport (ESCRT). EV uptake by target cells appears to occur most commonly by endocytosis or phagocytosis followed by membrane fusion within the endocytic compartment. An alternative pathway is direct membrane fusion at the cell surface.

**Table 1:** Summary of the differential properties of exosomes and microvesicles. ESCRT, endosomal sorting complex required for transport; MVB, multivesicular body

	Exosomes	Microvesicles
Alternative names	Extracellular vesicles	Extracellular vesicles, microparticles, shedding vesicles, ectosomes
Size	50–150 nm	100–1000 nm
Biogenesis	As ILVs in MVBs, followed by extracellular release by fusion of the MVBs with the plasma membrane	Direct shedding from the plasma membrane
Characteristic proteins	Tetraspanins (e.g., CD63, CD81, CD9), ESCRT components (ALIX, TSG101)	Integrins, selectins

### Pathophysiological relevance of redox-mediated EV signaling

EVs are actively involved in the pathophysiology of conditions that are associated with local or systemic inflammation and oxidative stress, such as unhealthy aging (also termed ‘inflammaging’), cancer, cardiovascular disease (CVD) and chronic lung diseases [13–16]. Several in vitro studies have shown that exposure of EV-secreting cells to pro-inflammatory or pro-oxidant conditions causes pathological changes in EV signaling [17, 18]. Moreover, chronic inflammatory diseases are associated with oxidative stress as well as elevated EV concentrations and altered EV composition, both of which can contribute to adverse biological effects of EVs [15, 19, 20]. Pharmacological modulation of EV formation may, therefore, be a promising treatment strategy for multiple chronic inflammatory conditions. However, most known targets for inhibiting EV release, including neutral sphingomyelinase 2 and the GTPase Rab27a [21, 22], are required for vital cellular processes such as lipid biosynthesis and intracellular membrane trafficking [23, 24]. Therefore, currently known strategies for EV inhibition are likely associated with off-target effects, hampering their application for the therapeutic modulation of EV signaling in vivo. An improved understanding of the mechanisms that modulate EV release under conditions of chronic inflammation and oxidative stress could allow identifying pharmacological compounds that only prevent pathological changes in EV signaling, but do not interfere with the physiological functions of EVs. Protein thiols can undergo reversible redox modifications. Thereby, they can act as switches in cellular redox signaling, and determine the cell’s response to changes in its redox environment [25, 26]. In this review article, we discuss how thiol modifications may be involved in EV formation and propose thiol protection as a novel strategy for preventing pathological changes in EV signaling.

## Redox regulation of protein thiols

This section provides general background information on how redox modifications of protein thiols affect protein function and, consequently, cellular functions. It aims to familiarize the reader with important principles and terminology in the field of redox-dependent thiol modifications before we discuss “Modulation of EV signaling by thiol modifications” in the next section.

Thiol groups in proteins are contributed by the amino acid cysteine. Most commonly, protein thiols exist either in a free, reduced form (Cys–SH), or oxidized to a disulfide with another thiol group of the same or another protein (Cys-S-S-Cys) [27]. During protein biosynthesis, disulfide bonds are introduced in a highly regulated manner. They are essential for correct three-dimensional protein structure and, consequently, for protein function. To assure correct protein folding, the position of cysteine residues within proteins is highly conserved [28]. Reduced thiols are nucleophilic and susceptible to electrophilic attacks because of their electron-rich sulfur atom [25]. Certain protein microenvironments polarize the S–H bond and thereby lower the acid dissociation constant (p*K*_a_) of thiol groups. This favors their deprotonation (i.e., the dissociation of H^+^), resulting in the formation of even more reactive thiolate anions (Cys-S^−^) [27]. This often occurs at active site cysteines, making them especially susceptible for redox modifications [29].

**Fig. 1:**
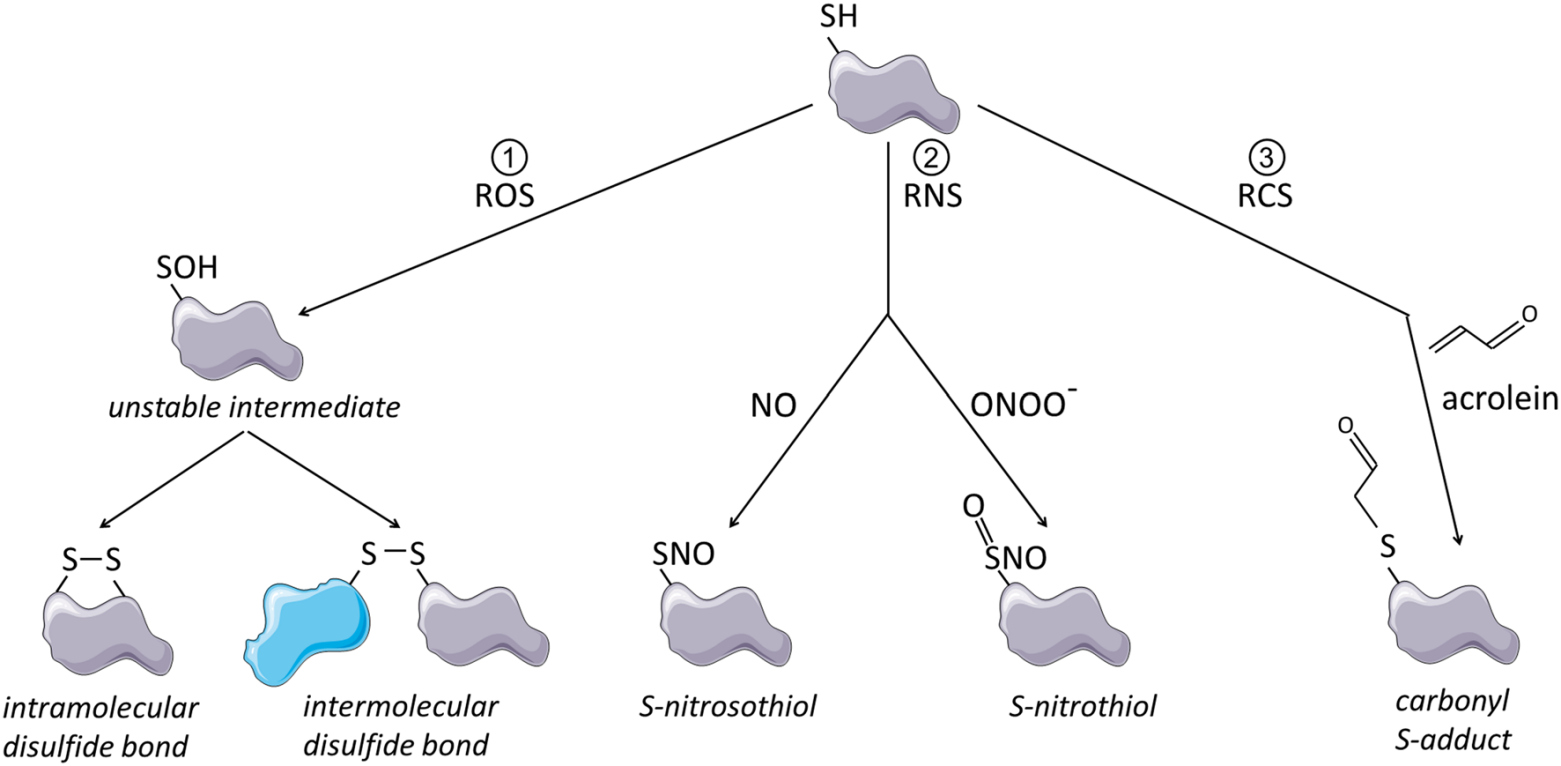
Schematic representation of thiol modifications by ROS, RNS and RCS. (1) Thiol oxidation by ROS leads to formation of an unstable cysteine sulfenic acid intermediate. This unstable intermediate can react with other thiol groups within the same or another molecule, which leads to formation of intramolecular or intermolecular disulfide bonds. (2) Thiol modification by RNS such as nitric oxide (NO) or peroxynitrite (ONOO^−^) leads to S-nitrosylation or S-nitration. (3) Thiol modification by RCS causes formation of relatively large and bulky carbonyl S-adducts as illustrated here for acrolein. This figure was created using Servier Medical Art

Electrophilic compounds that are prone to react with thiolates or thiols include reactive oxygen species (ROS), which can lead to the formation of ectopic disulfide bonds, reactive nitrogen species (RNS) which lead to S-nitrosylation or S-nitration (Cys-S-NO) and reactive carbonyl species (RCS) which result in adduct formation (Cys-S-R) [26] (illustrated in Fig. 1). ROS, RNS and RCS are present in environmental exposures, including cigarette smoke and vehicle emissions [30–32] and are also formed endogenously, for instance, during inflammation and lipid peroxidation [33–35]. Reaction of such oxidative stimuli with protein thiols can strongly affect protein conformation and functionality and cysteine-rich proteins often underlie redox regulation, especially those containing unpaired cysteines with free thiol groups [27]. The cell has a powerful antioxidant system at its disposal, which consists of glutathione and various antioxidant enzymes [36]. Up to moderate oxidant levels, this system is able to control and reverse thiol modifications by ROS and RCS, thereby allowing oxidative thiol modifications to act as transient and specific cell signaling events [36, 37]. Studies that investigate the regulation of protein function by redox sensitive thiols often apply electrophiles that covalently modify thiols, trapping them either transiently or irreversibly in an oxidized state [29]. Here, we will refer to these chemicals as thiol scavengers.

In the following sections, we discuss how thiol modifications may be involved in EV formation and how thiol protection may be used to prevent pathological changes in EV signaling.

## Modulation of EV signaling by thiol modifications

### Modulation of EV release

Intriguingly, it was reported as early as 1961 that treatment of various cells with thiol-reactive compounds induces blebbing of the plasma membrane [38]. In 1979, Scott and Maercklein found that these blebs were released into the cell culture media as 100 nm- to 10 µm-sized vesicles and could be isolated by centrifugation at 30,000×*g* [39]. The phenomenon was universal for cultured cells from different species and tissues, including fibroblasts, monocytes and macrophages and could be induced by a wide panel of RCS, including formaldehyde, *N*-ethyl-maleimide (NEM) and acrolein [39]. More recently, we and others have confirmed that treatment of various cell types with RCS or ROS enhances the release of EVs (for details, see Table 2) [40–43]. A number of studies have shown that both ROS-dependent and RCS-dependent EV induction are preventable by thiol-based antioxidants such as NAC [41–43], suggesting that thiol-reactivity is causally linked to EV induction. Yet, while Vatsyayan et al. proposed that RCS-induced EV release is mediated by secondary ROS generation, we found that only RCS, but not the ROS hydrogen peroxide, elicit increased EV release [40, 41]. Vatsyayan et al. and the studies that showed ROS-induced EV release detected EVs by direct flow cytometry for microvesicles (detection limit 300 nm) [40, 42, 43], whereas we used a combination of tunable resistive pulse sensing and bead-based flow cytometry to detect small EVs (85–250 nm) expressing exosome marker proteins [41]. Therefore, thiol modifications by RCS and ROS may differentially affect the release of microvesicles and exosomes. Moreover, it may depend on the cell type and its repertoire of proteins with redox sensitive thiols whether a certain stimulus does or does not trigger EV release. For instance, in the study by Vatsyayan et al., cell exposure to the RCS 4-hydroxy-2-nonenal enhanced the release of EVs by endothelial cells and fibroblasts, but not by monocytes [40].

**Table 2:** Experimental evidence for the involvement of protein thiols in the regulation of EV release

Study	Cell type	Exogenous thiol-reactive compounds	Exposure time	Endogenous thiol-reactive compounds	Thiol antioxidants	EV nomenclature	Effect of the thiol-reactive compounds on EV release	Summary of major findings
Belkin and Hardy [38]	Ascites cells, malignant cells, non-malignant cells	RCS (NEM; iodoacetamide) ROS (H_2_O_2_) Others (mercurial diuretics)	≤ 60 min	n.d.	None	Plasma membrane blebs	↑	All tested cell types respond to treatment with various extrinsic thiol-reactive compounds by plasma membrane blebbing
Scott et al. [39]	Fibroblasts, monocytes, myoblasts, etc.	RCS (e.g., formaldehyde, acrolein, NEM, iodoacetate)	30 min	n.d.	None	Plasma membrane blebs/vesicles	↑	All tested RCS, but not control compounds that do not react with thiols (e.g., succinimide), induce plasma membrane blebbing
Dachary-Prigent et al. [97]	Human platelets	RCS (NEM, diamide)	10 min	n.d.	None	Microparticles	(↓)	Platelet preincubation with RCS inhibits ionophore-induced calpain activity, PS externalization and EV release
Furlan-Freguia et al. [52]	Murine macrophages and smooth muscle cells	Thiol scavenger (DTNB)	30 min	ROS	NAC	Microparticles	↑	ATP stimulation results in endogenous ROS formation and release of procoagulant EVs. Blocking cell surface thiols with DTNB and ROS scavenging both prevent ATP-induced EV release
Vatsyayan et al. [40]	Human macrophages, coronary artery endothelial cells, fibroblasts	RCS (HNE, acrolein)	15 min–4 h	ROS	NAC, MPG	Microparticles	↑	In endothelial cells and fibroblasts, but not monocytes, extrinsic RCS induce procoagulant EVs. Intrinsic ROS generation and PS externalization are increased in all cell types and preventable by thiol protection
Novelli et al. [42]	Human alveolar and bronchial epithelial cells	ROS (H_2_O_2_)	20 h	n.d.	NAC	Microparticles	↑	Extrinsic ROS induce the release of procoagulant EVs by alveolar and bronchial epithelial cells, which is prevented by thiol protection
Carver et al. [43]	Human retinal pigment epithelial cells	ROS (H_2_O_2_)	2–24 h	n.d.	NACA	Microparticles	↑	Extrinsic ROS induce EV release. The EV release correlates with cellular apoptosis and is preventable by thiol protection
Szabó-Taylor et al. [47]	Human monocytes	None	90 min	n.d.	None	Extracellular vesicles	Not determined	Monocytes from pro-inflammatory conditions have increased exofacial thiols, but release EVs with decreased exofacial thiols. These EVs carry overoxidized proteins
Thom et al. [67]	Human and murine neutrophils and monocytes	None	2 h	ROS RNS	None	Microparticles	↑	Treatment of neutrophils with CO_2_ activates mitochondrial ROS generation and subsequent thiol-dependent activation of IP3 receptors. This causes calcium flux from the ER to the cytoplasm, and S-nitrosylation of actin, resulting in increased EV release
Benedikter et al. [41]	Human bronchial epithelial cells	RCS (acrolein) ROS (H_2_O_2_) Thiol scavengers (DTNB, bacitracin)	30 min–24 h	n.d.	NAC, GSH	Exosomes	↑ (RCS)/→ (ROS)	RCS and thiol scavengers, but not ROS deplete exofacial thiols and induce EV release. The EV induction by RCS can be prevented by thiol-protection

*AnxV* annexin V, *ATP* adenosine triphosphate, *CSE* cigarette smoke extract, *DTNB* 5,5-dithio-bis-(2-nitrobenzoic acid), *HCAEC* human coronary artery endothelial cells, *GSH* glutathione, *HNE* 4-Hydroxy-2-nonenal, *LPS* lipopolysaccharide, *NAC N*-acetyl-l-cysteine, *NACA N*-acetyl-l-cysteine amide, *NEM N*-ethylmaleimide, *MPG N*-(2-mercaptopropionyl)glycine, *PS* phosphatidylserine, *RCS* reactive carbonyl species, *RPE* retinal pigment epithelial cells, *TEM* transmission electron microscopy, *TNF-α* tumor necrosis factor α, *TF* tissue factor, *TRPS* tunable resistive pulse sensing

In some studies, ROS or RCS-induced microvesicle production was associated with a considerable amount of apoptotic cell death [23, 48], suggesting that microvesicle shedding in the response to thiol-reactive compounds may be due to cytotoxicity rather than being directly mediated by thiol modifications. However, we have found that the thiol scavengers 5,5-dithio-bis-(2-nitrobenzoic acid) DTNB and bacitracin induce EVs without affecting cell viability [29] and other mechanisms have been implied in ROS and RCS-dependent EV induction, as will be discussed later. While the effect of RNS on EV release is less well studied than that of ROS and RCS, there is evidence that nitric oxide (NO) negatively regulates EV release [44, 45]. Table 2 gives an overview of the experimental evidence for modulation of EV release by thiol-reactive compounds.

### Modulation of EV cargo and functions

Functional implications of EVs released under oxidative stress conditions have been described in detail elsewhere [18, 46]. Therefore, we will only discuss those studies that explicitly investigated EV functions related to oxidative thiol modifications. Szabó-Taylor et al. have exposed monocytes to pro-inflammatory conditions associated with oxidative stress and assessed the expression of the thiol-dependent redox enzyme peroxiredoxin 1 on the cells and their EVs [47]. While exofacial peroxiredoxin 1 was readily detectable on both, secreting cells and EVs, the over oxidized and enzymatically inactive form was exclusively enriched on the EVs [47]. This suggests that cells may release membrane proteins with oxidized thiol groups on EVs to maintain a reduced membrane status in oxidative environments. Similarly, the thiol groups of the cytosolic enzyme glyceraldehyde 3-phosphate dehydrogenase (GAPDH) become oxidized during red blood cell storage and the oxidized form of GAPDH is then released in an EV-associated manner [48]. This implies that intraluminal proteins with oxidized thiols may also be released in EVs as a protective mechanism.

However, the release of EVs under thiol-depleting conditions may not only confer cellular protection, as EV-associated oxidized proteins and phospholipids can serve as danger-associated molecular patterns and trigger inflammation [49]. Oxidative thiol modifications also appear to promote coagulation in an EV-dependent manner. A variety of thiol-depleting oxidative and pro-inflammatory conditions have been found to result in accumulation of prothrombotic EVs in vitro and in vivo [40, 42, 50–53]. The prothrombotic effect of these EVs has been ascribed to the phospholipid phosphatidylserine (PS) [42, 50, 53] and to EV-associated tissue factor (TF) [40, 42, 50, 52]. PS-rich membranes provide a negatively charged surface for the assembly of coagulation factors and thereby promote coagulation [54]. Since PS is considered a universal constituent of the outer leaflet of EV membranes [55], an increase in the number of secreted EVs should be sufficient to enhance PS-dependent coagulation. TF is the initiator of the extrinsic coagulation cascade [56]. Expression and activity of TF are both increased in EVs secreted by cells stimulated with thiol-reactive compounds [40, 42, 50, 52]. Intriguingly, the activity of TF increases when its free thiol groups are oxidized to form a disulfide bond [57], suggesting that redox modifications are important regulators of both, quantity and activity of EV-associated TF.

### Summary

Cell exposure to thiol-reactive compounds, particularly RCS and ROS, results in plasma membrane blebbing and increased release of EVs. EVs released in response to thiol modifications protect the secreting cell from oxidative damage, but also promote potentially harmful processes such as inflammation and coagulation.

## Molecular targets of thiol modifications that regulate EV release or EV uptake

**Fig. 2:**
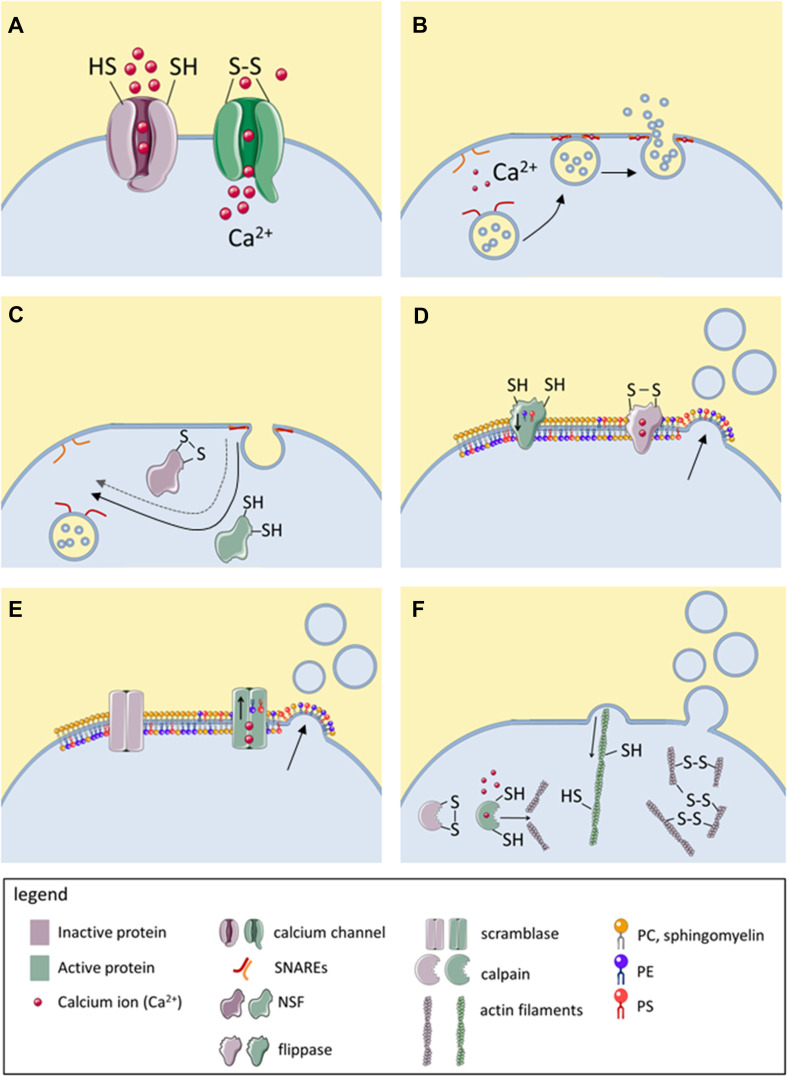
Known modulators of EV release that are directly or indirectly regulated by redox-sensitive thiols. Active proteins are represented in green and inactive proteins in purple. Disulfide bonds (–S–S–) in this figure are used representatively for all oxidative thiol modifications. **a** Several calcium channels become activated upon thiol oxidation, resulting in calcium influx and increased cytoplasmic calcium concentration. **b** Upon the thiol-dependent calcium influx, SNAREs mediate calcium-dependent fusion of MVBs with the plasma membrane, resulting in exosome release. **c** Reduced, but not oxidize NSF catalyzes the separation of v-SNAREs and t-SNAREs, allowing their recovery for repeated membrane fusion events. **d** Flippases ensure localization of PE and PS in the inner membrane leaflet. Upon thiol oxidation or upon thiol-dependent calcium influx, the enzymatic activity of flippase is inhibited, resulting in accumulation of PE and PS in the outer membrane leaflet and consequently, in membrane blebbing. **e** Upon thiol-dependent calcium influx, scramblase becomes activated, allowing PE and PS to diffuse to the outer membrane leaflet, enhancing membrane blebbing. **f** The actin cytoskeleton depends on reduced thiols for retracting membrane blebs. Oxidation of actin thiols causes depolymerization of actin filaments and impairs their functionality. Moreover, actin filaments can be degraded by calpains, cysteine proteases which are activated by cytoplasmic calcium but inactivated by thiol oxidation. NSF, *N*-ethylmaleimide sensitive factor; PC, phosphatidylcholine; PE, phosphatidylethanolamine; PS, phosphatidylserine SNARE, soluble NSF attachment protein receptor; t-SNARE, target membrane-associated SNARE; v-SNARE, vesicle-associated SNARE. This figure was created using Servier Medical Art

Although thiol modifications modulate the release and biological functions of EVs, relatively little is known about the thiol-bearing proteins that mediate these changes. In this section, various thiol-dependent mechanisms are presented that regulate either membrane fusion or blebbing and that are known or hypothesized to modulate EV release. These mechanisms are summarized in Table 3 and visualized in Fig. 2. Additionally, three recent publications are discussed in detail, which specifically imply modifications of cell surface-exposed thiols in EV release [41, 47, 52].

**Table 3:** Molecular targets with redox-sensitive thiol groups that regulate EV biology

Molecular target	Subcellular location	Mechanism of action	Active form	(Expected) Effect of oxidation/thiol blockage	Mechanism of EV release	References
Calcium channels (TRPA1, RyR, L-type channels, SERCA, IP3 receptors)	Transmembrane	Calcium influx into the cytoplasm inhibits flippases, activates SNARE-dependent membrane fusion and promotes calpain-and caspase-dependent cytoskeletal reorganization	Oxidized/adducted/nitrosylated	Increased EV release	Plasma membrane blebbing, fusion of MVE with plasma membrane	[60, 61, 67]
Calcium channels (SERCA, L-type channels, T-type channels)			Reduced	Decreased EV induction by activators of these channels		[60]
NSF	Intracellular	Recovers SNAREs for repeated rounds of membrane fusion	Reduced	Decreased EV release	Fusion of MVE with plasma membrane	[69, 72–74]
PDI family members	ER, cell surface	Unknown, may catalyze thiol-dependent conformational changes in fusion proteins	Reduced (reductase) Oxidized (oxidase)	Decreased (thiol blockage, DTNB)/increased (oxidation, ROS) EV release observed	Unknown, EVs can have microvesicle- or exosome-like properties	[52, 81, 83]
Thiol-rich fusion proteins (syncytin-1,-2, EFF-1, AFF-1)	Transmembrane	Mediate membrane fusion	Reduced	Decreased EV uptake	Involved in EV uptake (syncytin-1,-2), unknown (EFF-1, AFF-1)	[87, 109]
Phospholipid flippases	Transmembrane	Maintain conical phospholipids (PS, PE) in inner membrane leaflet	Reduced	Increased EV release	Plasma membrane blebbing	[89]
Actin cytoskeleton	Intracellular	Retraction of membrane blebs	Reduced	Increased EV release	Plasma membrane blebbing	[67, 95]
Calpains	Intracellular, can be membrane-associated	Degrade actin cytoskeleton and thereby prevent retraction of membrane blebs	Reduced	Decreased EV release	Plasma membrane blebbing	[97]
Cell surface thiols	Cell surface	Unknown	Reduced/oxidized (conflicting data)	Increased EV release?	Unknown, EVs can have microvesicles or exosome-like properties	[41, 47, 52]

*AFF-1* anchor cell fusion failure 1, *EFF-1* epithelial fusion failure 1, *EV* extracellular vesicle, *ER* endoplasmic reticulum, *IP3* inositol-1,3,5-triphosphate receptors, *MVE* multivesicular endosome, *NSF N*-ethylmaleimide-sensitive factor, *PDI* protein disulfide isomerase, *PE* phosphatidylethanolamine, *PS* phosphatidylserine, *RyR* ryanodine receptor, *SERCA* sarco/endoplasmic reticulum Ca^2+^-ATPase, *SNARE* soluble NSF attachment protein receptor, *TRPA1* transient receptor potential A1

### Redox sensitive calcium channels

Cytoplasmic calcium influx is a major inducer of both, microvesicle and exosome release, as it promotes membrane blebbing as well as fusion of MVBs with the plasma membrane [58, 59]. Intriguingly, several calcium channels bear redox-sensitive thiol groups and become activated upon their oxidation [60–64] (Fig. 2a; for details of the different calcium channels see Table 3). The nociceptor transient receptor potential ankyrin subtype 1 (TRPA1) is among the best studied calcium channels whose activity is modulated by thiol modifications [65]. Expressed by sensory neurons and other sensory cells, including epithelial cells [65], TRPA1 becomes activated upon covalent thiol oxidation by RCS or ROS [61, 66]. While a causal link between TRPA1 activation and induction of EV release has to our knowledge not been investigated, oxidative stimuli such as cigarette smoke and acrolein cause TRPA1 activation [63] and also enhance EV release [41]. Direct evidence that thiol-dependent calcium flux to the cytoplasm is associated with increased EV release stems from a study by Thom et al. The authors have shown that, ROS-dependent thiol oxidation of inositol-1,3,5-triphosphate (IP3) receptors triggers calcium flux from the endoplasmic reticulum to the cytoplasm and, consequently, EV release [67]. However, it should be noted that some calcium channels are inhibited, rather than activated by oxidative modification of their thiols [60]. Therefore, it may depend on the types of calcium channels expressed by a cell whether cytoplasmic calcium influx and, consequently, EV release is promoted or inhibited by cell exposure to thiol-reactive species.

Additionally to the direct effects of thiol modifications on EV release, the coming sections will also address how cytoplasmic calcium concentrations influence EV release.

### SNAREs and NSF

Soluble* N*-ethylmaleimide sensitive factor attachment protein receptors (SNAREs) mediate various intracellular membrane fusion events and are also involved in EV release [68–70]. In the presence of cytoplasmic calcium, e.g., upon activation of thiol-regulated calcium channels, vesicle-associated (v)-SNAREs and target membrane-associated (t)-SNAREs form a highly stable complex that can force two membranes together, resulting in membrane fusion (Fig. 2b) [71]. After SNARE-mediated fusion, the ATPase* N*-ethylmaleimide-sensitive factor (NSF) can translate the energy from ATP hydrolysis into a large conformational change that mechanically separates v-SNAREs and t-SNAREs, making them available for further membrane fusion events [71] (Fig. 2c). Importantly, activity of NSF depends on reduced thiols and can be inhibited by the thiol-reactive carbonyl* N*-ethyl-maleimide (NEM) [72]. SNAREs as well as NSF have been implicated in the fusion of MVBs with the plasma membrane, resulting in exosome release [69, 73, 74]. Taken together, under oxidative conditions, thiol-dependent calcium influx is expected to cause SNARE-dependent exosome release. Yet, oxidation of the free thiol group of NSF may prevent recovery of the SNARE proteins for subsequent membrane fusion events.

### The protein disulfide isomerase family and thiol-rich fusion proteins

The protein disulfide isomerase (PDI) family is a family of proteins with thiol-dependent oxidoreductase activity. The prototype family member PDI is expressed abundantly in most tissues and has two thioredoxin-like active sites (-Cys-XX-Cys-) [75]. Both active sites contain two cysteine residues, which catalyze various redox reactions, such as reduction of disulfide bonds, isomerization of disulfide bonds and oxidation of free thiols, by forming intermolecular disulfides with a substrate protein [76]. The redox state of PDI, and thus whether it is prone to catalyze oxidations or reductions, is controlled by other redox enzymes and by glutathione [75]. While most PDI is sorted to the endoplasmic reticulum (ER), significant amounts of PDI have also been detected associated with the plasma membrane, with the redox sites exposed on the cell surface [77], and secreted in an EV-associated form [52, 78, 79]. Importantly, PDI activity has been shown to be crucially involved in ATP-induced EV release by murine myeloid cells [52].

Several studies have implicated PDI family members as regulators of membrane fusion, making it conceivable that EV release and/or uptake can be mediated by PDI-dependent membrane rearrangements. For instance, sperm-egg fusion can be prevented by thiol scavengers and depends on expression of the PDI family member Erp57 on the sperm cell membrane and of the tetraspanins CD81 and CD9 on the egg cell membrane [80–82]. Notably, exosomal membranes are commonly associated with both tetraspanins and PDI, making it tempting to speculate that PDI may be involved in the fusion of tetraspanin-enriched membranes during EV biogenesis or uptake. Next to gamete fusion, the PDI family is also involved in the cell entry of several enveloped viruses, including retroviruses, whose biogenesis and cellular uptake bear striking resemblances to those of exosomes [83, 84]. PDI-catalyzed reduction of disulfide bonds in viral fusion proteins induces conformational changes which mediate fusion of the viral envelope with the host cell membrane [85, 86]. PDI may similarly regulate EV uptake by catalyzing thiol-disulfide exchange reactions in EV-borne fusion proteins. For instance, the fusion proteins syncytin-1 and syncytin-2 have been identified on EVs and proposed to mediate EV uptake by target cells [87]. The fusogenic activity of syncytins depends on several highly conserved cysteine residues [88], supporting the notion that they may be subject to thiol oxidoreductase regulation.

### Phospholipid flippases and scramblase

Phospholipid flippases and scramblase are enzymes that regulate the proportions of various phospholipids in the inner and outer leaflet of the cell membrane. Each membrane lipid has a characteristic shape due to the form of its head and the composition of its acyl tail. Therefore, the phospholipid composition of the inner and outer membrane leaflets determines membrane curvature. Under physiological conditions, phospholipid flippases assure the localization of the negatively charged phosphatidylserine (PS) and the conical phosphatidylethanolamine (PE) in the inner membrane leaflet. Importantly, flippase function depends on free thiol groups and is inhibited by thiol scavengers [89, 90]. This results in accumulation of PE and PS in the outer membrane leaflet and, consequently, in curving of the plasma membrane into blebs (Fig. 2d). Calcium influx, for instance triggered by oxidation of thiol-bearing calcium channels, also inhibits flippase function [91]. Furthermore, it stimulates the activity of scramblase, an enzyme that allows phospholipids to move along their concentration gradient from one membrane leaflet to the other (Fig. 2e) [91]. Together, calcium-dependent flippase inhibition and scramblase activation result in rapid accumulation of PS and PE in the outer leaflet and in bleb formation. Loss of membrane asymmetry is of direct relevance for microvesicle formation. In *C. elegans*, deletion of the PE-flippase TAT5 resulted in PE accumulation in the outer membrane leaflet and in large scale shedding of 100–200 nm sized microvesicles from the plasma membrane [92]. Additionally, a number of studies report that ROS or RCS-induced microvesicle production is associated with cellular PS externalization, which may be caused by flippase inactivation and/or scramblase activation [40].

### Retractive actin filaments and calpains

Under physiological conditions, membrane blebbing is counteracted by the retractive force of cytoskeletal actin filaments, whose activity depends on reduced thiols [93, 94] (Fig. 2e). Cytoskeletal inhibition by membrane-permeable thiol scavengers results in rapid and abundant shedding of plasma membrane-derived microvesicles (reviewed in [95]). The EVs induced by this treatment are very large (5–15 µm in diameter) and are thought to reflect the cytosolic and plasma membrane composition of their cells of origin [39, 95]. However, such large EVs rarely occur under physiological conditions. Thus, it remains to be elucidated whether blocking of cytoskeletal free thiols contributes to normal EV biogenesis. Thom et al. reported that exposure of neutrophils to CO_2_ results in increased activity of inducible nitric oxide synthase (iNOS) and, consequently, in S-nitrosylation of actin. Abrogation of actin S-nitrosylation by UV-light prevented the EV induction, suggesting that the S-nitrosylation was required for CO_2_-induced EV release. Intriguingly, the authors found that S-nitrosylated actin was associated with phospholipid flippase and PDI, which may further contribute to thiol-dependent regulation of EV release as discussed above.

Retraction of membrane blebs is additionally regulated by calpains, cysteine proteases that degrade actin filaments. Calcium-dependent activation of calpains prevents retraction of membrane blebs and promotes microvesicle formation [91, 96]. However, while calpains are activated by calcium, they become inactivated when their free thiols are oxidized. Therefore, calpain-dependent microvesicle release may be enhanced by membrane impermeable thiol-reactive compounds that activate calcium influx channels at the cell surface, but inactivated by thiol-reactive compounds that enter the cell and oxidize the active site thiols of calpains. Congruently, Dachary-Prigent et al. have shown that the membrane-permeable RCS *N*-ethylmaleimide and diamide inhibit calpain function and thereby prevent the release of platelet EVs in response to the calcium ionophore A23187 [97].

### Cell surface-exposed thiols

A number of recent studies have proposed that the redox state of cell surface-exposed thiols is involved in the regulation of EV release [41, 47, 52]. Firstly, Furlan-Freguia et al. have shown that stimulation of murine macrophages with the danger-associated molecular pattern (DAMP) ATP results in P2X7 receptor-dependent upregulation of free thiols at the cell surface and in ROS-dependent release of thiol-rich microvesicles [52]. Cell pre-treatment with the membrane impermeable thiol-scavenger DTNB, as well as inhibition of PDI reductase activity (using the anti-PDI clone RL90) prevented the microvesicle induction in response to ATP [52]. In contrast, inhibition of PDI oxidase activity (using the anti-PDI clone 34) directly induced increased cell surface thiols and thiol-rich microvesicle release [52]. A possible interpretation of these findings is that PDI may maintain thiols in an oxidized state under control conditions, thereby preventing microvesicle formation. Upon ATP exposure, PDI activity may then shift from oxidase to reductase activity, resulting in the appearance of free cell surface thiols and subsequent ROS-dependent induction of thiol-rich microvesicles. Similarly to Furlan-Freguia et al., Szabó-Taylor et al. have found that stimulation of human monocytes with pro-inflammatory stimuli, namely lipopolysaccharide (LPS) or tumor necrosis factor (TNF)-α, caused an upregulation of cell-surface-exposed thiols [47]. However, the EVs that these cells secreted were poor in exofacial thiols [47], in contrast to the thiol-rich EVs observed by Furlan-Freguia et al. While Szabó-Taylor et al. did not assess whether there was a quantitative change in EV release when monocytes were stimulated with LPS or TNF-α, they did hypothesize that the shedding of thiol-poor EVs may be a protective mechanism to maintain the cell surface in a reduced state [47].

Data from our group further corroborates that the redox state of exofacial thiols regulates EV release [41], although part of our results appear to disagree with findings of the two other groups. We found that treatment of airway epithelial cells with the RCS acrolein, but not with the ROS H_2_O_2_ causes depletion of cell surface thiols. Acrolein as well as the membrane impermeable thiol scavengers DTNB and bacitracin triggered increased release of small EVs expressing the exosome markers CD63 and CD81, whereas H_2_O_2_ had no measurable effect on EV release. The EV induction appeared to be directly caused by the depletion of cell surface thiols rather than being associated with the cell’s adaptive antioxidant response because EV induction required continuous presence of the RCS, whereas a transient RCS-stimulation was sufficient to induce upregulation of the cellular antioxidant glutathione [41]. In our study, neither the anti-PDI clone RL90 nor the PDI-inhibitor rutin affected basal or RCS-induced EV release. Although we were unable to identify a specific exofacial target protein of thiol modifications, we could conclude that depletion of cell surface thiols is sufficient to elicit an increased EV release in airway epithelial cells.

Although all three studies provide evidence that cell surface thiols may be crucially involved in the regulation of EV release, a number of discrepancies remain. While Furlan-Freguia et al. argue that an increase in the number of cell-surface thiols is required for EV induction [52], we found that depletion of exofacial thiols enhances EV release [41]. Another discrepancy is that EVs released by cells with increased cell-surface thiols were rich in exofacial thiols in the study of Furlan-Freguia et al. [52], while they were poor in exofacial thiols in the study of Szabó-Taylor et al. [47]. Additional research is required to reveal whether the differences between studies are due to the stimuli, the cell types, the EV subpopulations or the exposure times that were investigated. It has to be noted that oxidative processes were involved in the cell surface thiol-dependent EV release in all three studies. Thus, initial upregulation of reduced cell surface thiols may be a prerequisite for subsequent oxidant-dependent thiol modifications and EV induction. It should be investigated whether the amount of cell surface thiols regulates EV release via one or more specific thiol-bearing proteins, or whether a more general mechanism is involved, such as disulfide cross-linking of exofacial thiol-bearing proteins by ROS or formation of bulky adducts by RCS, both of which may influence membrane curvature.

## Thiol protection to prevent EV modifications: therapeutic implications

As thiol modifications appear to modulate the formation and functions of EVs upon cell exposure to pro-oxidant conditions, thiol protection may be a promising strategy to prevent detrimental changes in EV signaling under such conditions. Indeed, several thiol-bearing small molecules, such as NAC; NACA and glutathione are able to prevent EV induction by a variety of ROS, RCS and pro-inflammatory stimuli, likely by scavenging thiol-reactive compounds and preventing them from reacting with cellular thiols [41–43, 52, 53, 98–100]. NAC also inhibits EV-associated release of TF and PS by cells exposed to oxidant conditions and consequently decreases the procoagulant potential of EVs [42, 52, 53]. It may also prevent EV-dependent secretion of pro-inflammatory molecules [98], although this has been less well studied. Importantly, NAC treatment appears to restore EV secretion, composition and functions to the level observed for unexposed cells, rather than completely inhibiting EV signaling. Thus, NAC may specifically prevent oxidant-induced detrimental changes in EV signaling without interfering with the physiological functions of EVs.

In lung disease, particularly chronic obstructive pulmonary disease (COPD), NAC is currently used as a mucolytic. According to recent meta-analyses, NAC is associated with improved small airway function and decreased exacerbation frequency in this target group when administered orally at ≥ 1200 mg/day [101–103]. Importantly, it has been proposed that these clinical benefits can at least partly be attributed to antioxidant and anti-inflammatory properties of NAC or its thiol-bearing metabolites rather than to the mucolytic activity alone [102, 104]. Additional research could reveal whether prevention of cellular thiol modifications and subsequent changes in EV signaling contribute to this alternative mechanism of action. Importantly, NAC has recently been proposed to be of clinical benefit in other conditions that are also hallmarked by inflammation and oxidative stress, such as insulin resistance and possibly neurological disorders [105, 106]. There is even early stage evidence suggesting an anti-thrombotic effect of NAC treatment [104, 107, 108]. This is in line with the observation that NAC prevents the release of procoagulant EVs in the response to oxidative thiol modifications [42, 52, 53]. Translational studies are required to elucidate whether inhibition of thiol-dependent EV modifications contributes to the clinical benefit of NAC in COPD patients and to determine whether additional target groups may benefit from NAC treatment due to this mechanism of action.

## Conclusion

Taken together, protein thiols play a crucial role in the modulation of membrane fusion and membrane blebbing. Thereby, they regulate EV release and, possibly, uptake. EV release under thiol-depleting conditions may have evolved as a beneficial adaptive response to cellular oxidative stress. However, these EVs may also exert detrimental pro-inflammatory and prothrombotic effects. Additional research is required to establish the importance of thiols in EV biology and to identify the molecular mechanisms that mediate the thiol-dependent regulation of EV-related membrane rearrangements. Many known inhibitors of EV signaling interfere with vital cellular processes, making them unsuitable for the therapeutic modulation of EV signaling. Thiol-containing antioxidants such as NAC counteract the induction of EVs by pro-oxidant stimuli in vitro. Clinical studies are required to investigate whether inhibiting the release of pro-inflammatory and procoagulant EVs contributes to the therapeutic benefit of NAC in conditions of chronic inflammation and oxidative stress. Additionally, future research should focus on identifying the specific thiol-dependent mechanisms that are involved in the regulation of EV release and uptake, as these may be promising targets for specific pharmacological modulation of EV signaling.

## Abbreviations

AFF-1: Anchor cell fusion failure 1
AnxV: Annexin V
ATP: Adenosine triphosphate
CSE: Cigarette smoke extract
DTNB: 5,5-Dithio-bis-(2-nitrobenzoic acid)
EFF-1: Epithelial fusion failure 1
ER: Endoplasmic reticulum
ESCRT: Endosomal sorting complex required for transport
EV: Extracellular vesicle
GAPDH: Glyceraldehyde 3-phosphate dehydrogenase
GSH: Glutathione
HCAEC: Human coronary artery endothelial cells
HNE: 4-Hydroxy-2-nonenal
ILV: Intraluminal vesicle
LPS: Lipopolysaccharide
MPG: *N*-(2-Mercaptopropionyl)glycine
MVE: Multivesicular endosome
NAC: *N*-acetyl-l-cysteine
NACA: *N*-acetyl-l-cysteine amide
NEM: *N*-ethylmaleimide
NSF: *N*-ethylmaleimide sensitive factor
PDI: Protein disulfide isomerase
PE: Phosphatidylethanolamine
PS: Phosphatidylserine
RCS: Reactive carbonyl species
ROS: Reactive oxygen species
RPE: Retinal pigment epithelial cells
PRDX1: Peroxiredoxin 1
RyR: Ryanodine receptor
SERCA: Sarco/endoplasmic reticulum Ca^2+^-ATPase
SNAP: Soluble NSF attachment protein
SNARE: SNAP receptor
TEM: Transmission electron microscopy
TF: Tissue factor
TNF-α: Tumor necrosis factor α
TRPA1: Transient receptor potential A1
t-SNARE: Target membrane-associated SNARE
TRPS: Tunable resistive pulse sensing
VAMP7: Vesicle-associated membrane protein 7
v-SNARE: Vesicle-associated SNARE
